# Human *Clostridium difficile* infection caused by a livestock-associated PCR ribotype 237 strain in Western Australia

**DOI:** 10.1099/jmmcr.0.005062

**Published:** 2016-08-30

**Authors:** Alan M. Mc Govern, Niki F. Foster, Lynette A. Pereira, Daniel R. Knight, Briony Elliott, Barbara J. Chang, Thomas V. Riley

**Affiliations:** ^1^​ School of Pathology and Laboratory Medicine, The University of Western Australia, 6009 Nedlands, Western Australia; ^2^​ Department of Microbiology, Path West Laboratory Medicine, Queen Elizabeth II Medical Centre, 6009 Nedlands, Western Australia; ^3^​ Department of Infectious Diseases, Royal Perth Hospital, 6000 Perth, Western Australia

**Keywords:** *Clostridium difficile* infection (CDI), zoonosis, diarrhoea, PCR ribotyping, antibiotics, metronidazole

## Abstract

**Introduction:**

*Clostridium difficile* infection (CDI) is a significant gastrointestinal disease in the developed world and increasingly recognised as a zoonotic infection. In North America and Europe, the PCR ribotype (RT) 078 strain of *C. difficile* is commonly found in production animals and as a cause of disease in humans although proof of transmission from animals is lacking. This strain is absent in Australian livestock. We report a case of human CDI caused by a strain of *C. difficile* belonging to known Australian livestock-associated RT 237.

**Case presentation:**

A young male was admitted for multiple trauma following a motor vehicle accident and placed on piperacillin/tazobactam for pneumonia. After 4 days of treatment, he developed symptoms of CDI, which was confirmed in the laboratory. His symptoms resolved after 6 days of intravenous metronidazole. The strain of *C. difficile* isolated was identified as RT 237, an unusual RT previously found in with several Western Australia piggeries.

**Conclusion:**

This case of CDI caused by an unusual livestock-associated *C. difficile* RT 237 supports the hypothesis of zoonotic transmission. The case highlights the potential of livestock to act as reservoir for *C. difficile* and the need for continued surveillance of CDI in both human and animal populations.

## Introduction

*Clostridium difficile* is an anaerobic, spore-forming, Gram-positive bacillus that acts as an opportunistic pathogen causing diarrhoea and sometimes life-threatening conditions such as toxic megacolon and pseudomembranous colitis. Since the early 2000s, the number of cases of *C. difficile* infection (CDI) worldwide has increased dramatically, particularly in North America and Europe. In the USA alone, CDI has been identified as the major healthcare-associated infection with half a million patients infected and costing at least US$1 billion annually (Lessa *et al.*, 2015). An increasing number of infections are caused by strains belonging to PCR ribotypes (RTs) that are common to both humans and animals, the most prominent of which is RT 078 (Goorhuis *et al.*, 2008). Direct transmission of RT 078 between pigs and humans has been postulated in the Netherlands (Knetsch *et al.*, 2014) but never conclusively proven. In Australia, RTs associated with major outbreaks overseas such as RT 027 and RT 078 are rare; however, overall rates of CDI across Australia have increased recently (Slimings *et al.*, 2014). While RT 078 has not been detected in Australian livestock, a variety of other RTs has been isolated from production animals, including cattle, pigs and sheep (Knight & Riley, 2013; Knight *et al.*, 2015c; Knight *et al.*, 2013; Moono *et al.*, 2016; Squire *et al.*, 2013). In several piggeries in Western Australia (WA), an unusual RT of *C. difficile* (RT 237) is endemic (Squire *et al.*, 2013; Moono *et al.*, 2016). This report describes the isolation and characterisation of a *C. difficile* RT 237 strain in a case of human infection, initially identified in a cross-sectional study of CDI epidemiology in WA (Foster *et al.*, 2014).

## Case report

A 19-year-old Caucasian male was admitted to Bunbury Regional Hospital (BRH) in WA during February 2012 for multiple trauma following a motor vehicle accident. The patient, a New Zealand national, was in Australia working as a dairy farmer. He had been hospitalised at BRH 3 months earlier for a crush injury of his hand. He had no known allergies and his only other medical history was of mild asthma requiring occasional inhaled salbutamol. He was transferred from BRH to the State Trauma Unit at Royal Perth Hospital via the Royal Flying Doctor Service.

Initial computerised tomography and X-ray scans revealed a minor mandibular fracture, two severe distal phalangeal fractures and extensive pelvic fracturing with associated haematoma. Haemoperitoneum and lacerations to the skin, right kidney and bladder were also found. On admission, the patient was placed on 4.5 g intravenous (IV) piperacillin/tazobactam three times daily for 6 days to prophylax against infection complicating the pelvic and abdominal trauma. Pelvic injuries were initially managed conservatively, but on the second day of his admission, after worsening abdominal pain, he underwent an exploratory laparotomy. At the procedure , a bladder injury was noted and repaired. On the same day, he also underwent surgical debridement of the finger wounds. Mandibular fractures were managed conservatively.

On the fourth day of admission, the patient developed a fever with a temperature of 38.4 °C and associated tachycardia and tachypnoea. He began to exhibit abdominal distension and frequent bowel motions with loose stools. Blood cultures taken at this time were negative. At 7 days post-admission, IV piperacillin/tazobactam was ceased. The patient underwent an open reduction and internal fixation of the pubic symphysis and was commenced on IV cephazolin 1 g 6 hourly, as peri-operative prophylaxis for 48 h. His fevers continued while his bowel motions became more frequent and CDI was suspected. A stool sample was collected at 9 days post-admission for standard faecal microscopy and culture. At 10 days post-admission, he was noted to have pus discharging from the urethra and was recommenced on piperacillin/tazobactam 4.5 g IV three times daily for 5 days for treatment of presumed urinary tract infection. There was no significant pathogen identified from a urethral swab taken at the time.

PCR testing (BD GeneOhm™ Cdiff Assay, BD Diagnostics) of the stool specimen detected the presence of the *C. difficile* toxin B gene (*tcdB*). Vancomycin-resistant *Enterococcus* was also detected but *Aeromonas*, *Campylobacter*, *Norovirus*, *Salmonella*, *Shigella* and intestinal parasites were not. His haematological profile at this time showed thrombocytosis (628×10^9^ l^−1^) [normal range, 150 to 450×10^9^ l^−1^], neutrophilia (12.03×10^9^ l^−1^) [normal range, 2 to 7.5×10^9^ l^−1^], low haemoglobin (87 g l^−1^) [normal range, 115 to 165 g l^−1^] and hypo-albuminaemia (27 g l^−1^) [normal range, 35 to 45 g l^−1^]. Based on these laboratory results, the patient immediately began a 10 day course of metronidazole 400 mg IV three times daily and was placed under contact precautions. Ten days post-admission, the patient developed a deep infection complicating the pelvic plate. At this time, he was recommenced on piperacillin/tazobactam empirically. He underwent debridement of the wound on day 14 and day 19 of the admission, and he underwent an exchange of the pelvic plate on day 21 of his admission. No specimens for culture were obtained from the wound. The patient continued on piperacillin/tazobactam for 18 days, after which time he developed a presumed delayed hypersensitivity reaction to piperacillin/tazobactam (rash fever, eosinophilia and liver function derangement) and was subsequently changed to IV cephazolin 2 g 8 hourly. After 6 days of metronidazole treatment (16 days post-admission), his fever and CDI symptoms had subsided. A faecal specimen collected 23 days post-admission was PCR negative for the toxin B gene and the patient was discharged to the physical rehabilitation unit after a total of 46 days as an in-patient. The patient completed 6 weeks of IV antimicrobial therapy for the infection of the pelvic plate, after which time he continued on oral cephalexin, with the plan for this to continue this until the pelvic plate could be definitively removed. No further episodes of CDI were noted.

## Investigations

**Fig. 1.**
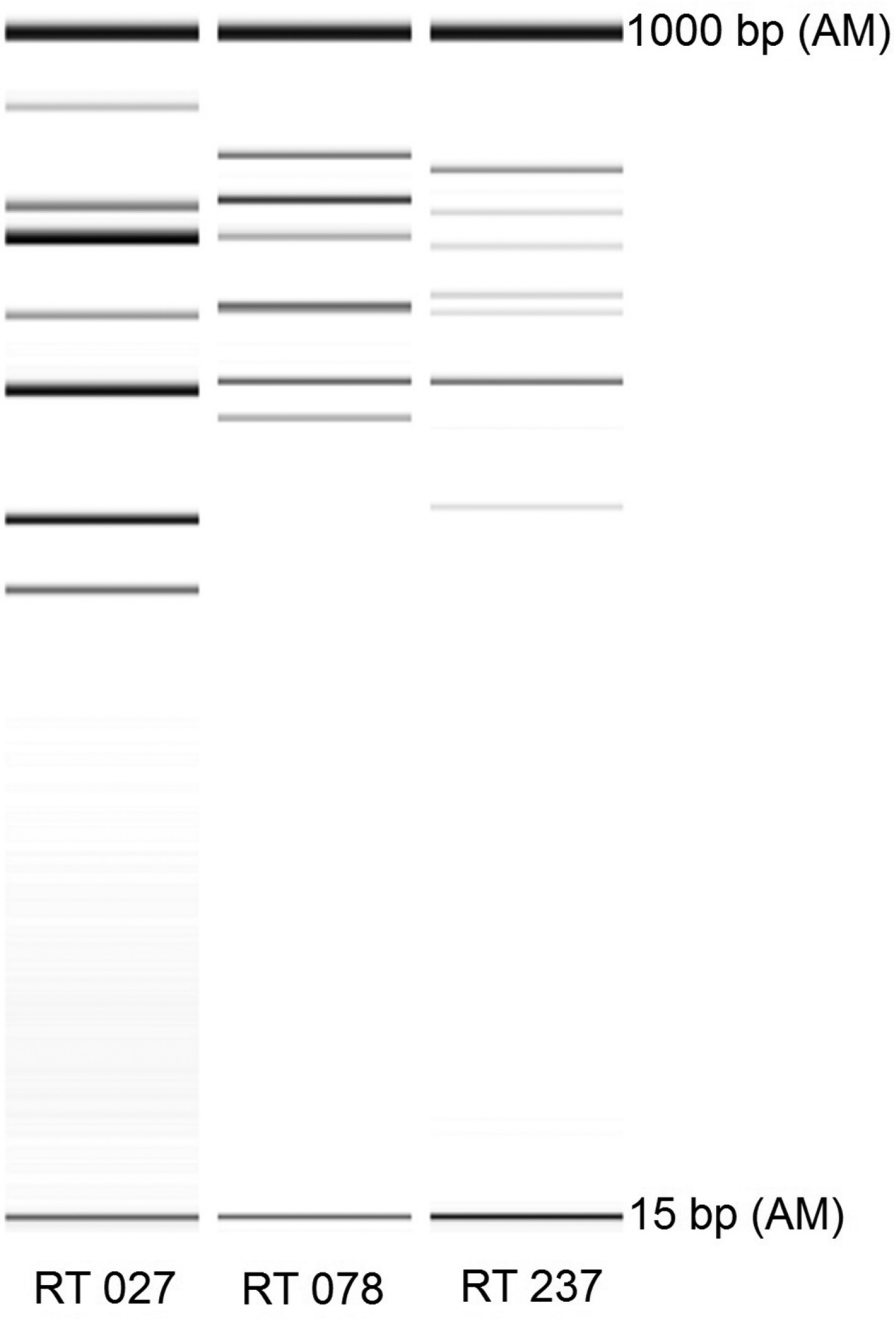
PCR ribotyping (RT) pattern of *C. difficile* RTs 027, 078 and 237 when visualised by capillary electrophoresis. AM, alignment marker.

*C*. *difficile* was cultured from the PCR-positive stool specimen as previously described (Boseiwaqa *et al.*, 2013); the strain isolated was designated WA 1016. PCR ribotyping and PCR detection of the genes encoding toxin A (*tcdA*), toxin B (*tcdB*) and the binary toxin (CDT) component genes (*cdtA*, *cdtB*) were performed as described previously (Knight *et al.*, 2013). WA 1016 gave a PCR ribotyping pattern consistent with RT 237 (Fig. 1) and was positive for *tcdB* and both CDT genes but negative for *tcdA* (A^−^B^+^CDT^+^), a toxin profile also consistent with *C. difficile* RT 237. Antimicrobial susceptibility testing was performed using the agar incorporation method as described by Clinical and Laboratory Standards Institute (CLSI) (CLSI, 2011; CLSI, 2013). All clinical breakpoints were provided by CLSI (CLSI, 2013) with the exception of those for vancomycin and rifaximin that were recommended by the European Committee on Antimicrobial Susceptibility Testing [EUCAST (2016) and O'Connor *et al.* (2008), respectively]. WA 1016 was resistant to trimethoprim but susceptible to vancomycin, metronidazole, rifaximin, clindamycin, erythromycin, moxifloxacin and piperacillin/tazobactam. The fidaxomicin minimum inhibitory concentration of WA 1016 was 0.008 mg l^−1^ (no clinical breakpoints for fidaxomicin are currently available). The antimicrobial susceptibility profile of WA 1016 matched those typical of animal RT 237 isolates (Knight & Riley, 2016).

## Discussion

The epidemiology of CDI is complex and ever-changing, with recent evidence pointing to genetically diverse sources of infection (Eyre *et al.*, 2013) and possible zoonotic transmission in Europe (Knetsch *et al.*, 2014). The potential for zoonotic transmission of *C. difficile* in Australia has also been suggested (Knight *et al.*, 2015b). In Australian livestock, the molecular epidemiology of *C. difficile* is different from that seen elsewhere in the world. The strain most commonly associated with production (food) animals in the global setting is RT 078 and this strain is not found in Australian livestock, although many related strains also belonging to phylogenetic clade 5 are present (Knight *et al.*, 2013). In Australian piglets, RTs 014 and 033 were the most common RTs recovered, followed by RTs 281 and 237 (Knight *et al.*, 2015c). In Australian veal calves slaughtered at 5–7 days old, the most frequently encountered RTs were RTs 127 and 033 followed by RTs 056 and 126 (Knight *et al.*, 2013). *C. difficile* RT 237 was absent in veal calves (Knight *et al.*, 2013). Crossover between animal and human RTs of *C. difficile* in Australia appears to be occurring with RTs 014 and 056 being common RTs found in both humans and animals (Foster *et al.*, 2014); however, this needs to be confirmed with whole genome sequencing. The high prevalence of RT 033 in livestock does not seem to be mirrored in humans; however, the absence of toxin A and B production in this strain may preclude its detection in many diagnostic laboratories (Androga *et al.*, 2015).

*C. difficile* RT 237 belongs to toxinotype XXXI and is missing a large portion of the 5′ end of the *tcdA* gene yet retains an intact *tcdB* gene (Rupnik & Janezic, 2016). Certain diagnostics tests reliant on detection of the missing fragment of *tcdA*, such as the Illumigene^®^*C. difficile* test (Meridian Bioscience), will also not detect RT 237 (Androga *et al.*, 2015). Diagnostic issues such as these are compounded by recent trends away from culture and greater reliance on molecular tests (Burnham & Carroll, 2013). *C. difficile* RT 237 is an unusual ribotype rarely seen as a cause of disease in humans in Australia. This is in marked contrast with RT 078, which is commonly detected in both humans and animals outside Australia (Goorhuis *et al.*, 2008). Interestingly, RT 078 infections in Australian patients still occur (Knight *et al.*, 2015a) in the absence of overseas travel suggesting another reservoir of infection, perhaps imported food. Since routine surveillance began in WA in 2011, only six other cases of human CDI involving RT 237 have been recorded (TV Riley *et al.*, unpublished data). However, *C. difficile* RT 237 has been isolated from WA piggeries for many years and remains highly prevalent (Moono *et al.*, 2016; Squire *et al.*, 2013). To our knowledge, RT 237 has not been reported elsewhere in the world and was not mentioned in a recent publication describing strains of *C. difficile* isolated from animal populations around the world (Janezic *et al.*, 2014). To what extent that the lack of *C. difficile* RT 237 infections described in other parts of the world is due to detection issues remains unclear.

The source of infection in our case is difficult to determine. As symptoms began after 48 h of admission to a healthcare facility, the case was classified as hospital-associated healthcare facility onset according to McDonald *et al*. (2007). No cases of RT 237 had occurred at BRH prior to the patient’s admission. The apparently exclusive distribution of *C. difficile* RT 237 in production animals in WA (Knight *et al.*, 2015c; Moono *et al.*, 2016; Squire *et al.*, 2013) is suggestive of transmission within the state. The patient lived in shared accommodation in a rural area working as a dairy farmer. RT 237 was not isolated from dairy (veal) calves in a previous Australian surveillance study (Knight *et al.*, 2013); however, this study did not include WA calves. *C. difficile* RT 237 has been isolated from several piggeries in WA and there is pig rearing in the region of WA where the patient lived. Comprehensive surveillance of *C. difficile* in livestock is not in place nor is it likely to be in the near future, making it difficult to get a complete picture of the distribution of this ribotype. We recently isolated *C. difficile* RT 237 from vegetables sold in several grocery stores in WA (Lim, Foster & Riley., unpublished data). This suggests contamination of vegetables by piggery effluent. Possible food-borne transmission of another clade 5 strain, RT 126, common in Australian cattle, has recently been reported (Knight *et al.*, 2015b). The potential of contaminated food as a route of transmission of *C. difficile* has been gaining more attention (Gould & Limbago, 2010; Rupnik, 2007; Weese, 2010). Additionally, the role of the broader environment as a reservoir for *C. difficile* requires further investigation. There are difficulties in studying transmission of *C. difficile* outside the hospital; however, data from the limited number of surveys performed so far suggest that *C. difficile* is present widely in soil and water (Al Saif & Brazier, 1996).

This report demonstrates that *C. difficile* RT 237, an animal strain of *C. difficile* reported only in WA, can be associated with human infection. This finding supports our previous observation that *C. difficile* RT 237 caused significantly greater weight loss in mice than *C. difficile* RT 078 due to the production of a variant toxin (Squire *et al.*, 2013) and highlights the potential risk that animals carrying this strain may pose to humans. Surveillance of animal populations is needed to clarify the relationship between livestock-associated *C. difficile* and human CDI. Ultimately, the promotion of a dialogue between physicians, veterinarians and scientists in the development of a One Health approach will be essential to control CDI.

## References

[R1] Al Saif N. Brazier J. S.(1996). The distribution of *Clostridium difficile* in the environment of South Wales. J Med Microbiol 45 133–137. 10.1099/00222615-45-2-133 8683549

[R2] Androga G. O. McGovern A. M. Elliott B. Chang B. J. Perkins T. T. Foster N. F. Riley T. V.(2015). Evaluation of the Cepheid Xpert *C. difficile*/Epi and meridian bioscience illumigene *C. difficile* assays for detecting *Clostridium difficile* ribotype 033 strains. J Clin Microbiol 53 973–975. 10.1128/JCM.03297-14 25520452 PMC4390624

[R3] Boseiwaqa L. V. Foster N. F. Thean S. K. Squire M. M. Riley T. V. Carson K. C.(2013). Comparison of ChromID *C. difficile* agar and cycloserine-cefoxitin-fructose agar for the recovery of *Clostridium difficile*. Pathology 45 495–500. 10.1097/PAT.0b013e3283632680 23846295

[R4] Burnham C. A. Carroll K. C.(2013). Diagnosis of *Clostridium difficile* infection: an ongoing conundrum for clinicians and for clinical laboratories. Clin Microbiol Rev 26 604–630. 10.1128/CMR.00016-13 23824374 PMC3719497

[R5] CLSI(2011). Methods for Antimicrobial Susceptibility Testing of Anaerobic Bacteria. Seventh Edition: Approved Standard M11-A7. Wayne, PA. USA: CLSI.

[R6] CLSI(2013). Performance Standards for Antimicrobial Susceptibility Testing: Twenty-third Informational Supplement M100-S23. Wayne, PA, USA: CLSI.

[R7] EUCAST(2016). *Clinical Breakpoint Tables for Interpretation of MICs and Zone**Diameters, Version 66.0* [Online] Available: http://www.eucast.org/clinical_breakpoints/ [Accessed 21/04/20116].

[R8] Eyre D. W. Cule M. L. Wilson D. J. Griffiths D. Vaughan A. O'Connor L. Ip C. L. Golubchik T. Batty E. M.(2013). Diverse sources of *C. difficile* infection identified on whole-genome sequencing. N Engl J Med 369 1195–1205. 10.1056/NEJMoa1216064 24066741 PMC3868928

[R9] Foster N. F. Collins D. A. Ditchburn S. L. Duncan C. N. Van Schalkwyk J. W. Golledge C. L. Keed A. B. Riley T. V.(2014). Epidemiology of *Clostridium difficile* infection in two tertiary-care hospitals in Perth, Western Australia: a cross-sectional study. New Microbes New Infect 2 64–71. 10.1002/nmi2.43 25356346 PMC4184660

[R10] Goorhuis A. Bakker D. Corver J. Debast S. B. Harmanus C. Notermans D. W. Bergwerff A. A. Dekker F. W. Kuijper E. J.(2008). Emergence of *Clostridium difficile* infection due to a new hypervirulent strain, polymerase chain reaction ribotype 078. Clin Infect Dis 47 1162–1170. 10.1086/592257 18808358

[R11] Gould L. H. Limbago B.(2010). *Clostridium difficile* in food and domestic animals: a new foodborne pathogen? Clin Infect Dis 51 577–582. 10.1086/655692 20642351

[R12] Janezic S. Zidaric V. Pardon B. Indra A. Kokotovic B. Blanco J. L. Seyboldt C. Diaz C. R. Poxton I. R.(2014). International *Clostridium difficile* animal strain collection and large diversity of animal associated strains. BMC Microbiol 14. 10.1186/1471-2180-14-173 24972659 PMC4100527

[R13] Knetsch C. W. Connor T. R. Mutreja A. Van Dorp S. M. Sanders I. M. Browne H. P. Harris D. Lipman L. Keessen E. C.(2014). Whole genome sequencing reveals potential spread of *Clostridium difficile* between humans and farm animals in the Netherlands, 2002 to 2011. Euro Surveill 19 20954. 10.2807/1560-7917.ES2014.19.45.20954 25411691 PMC4518193

[R14] Knight D. R. Riley T. V.(2013). Prevalence of gastrointestinal *Clostri- dium difficile* carriage in Australian sheep and lambs. Appl Environ Microbiol 79 5689–5692. 10.1128/AEM.01888-13 23851101 PMC3754155

[R15] Knight D. R. Thean S. Putsathit P. Fenwick S. Riley T. V.(2013). Cross-sectional study reveals high prevalence of *Clostridium difficile* non-PCR ribotype 078 strains in Australian veal calves at slaughter. Appl Environ Microbiol 79 2630–2635. 10.1128/AEM.03951-12 23396338 PMC3623178

[R16] Knight D. R. Giglio S. Huntington P. G. Korman T. M. Kotsanas D. Moore C. V. Paterson D. L. Prendergast L. Huber C. A.(2015a). Surveillance for antimicrobial resistance in Australian isolates of *Clostridium difficile*, 2013-14. J Antimicrob Chemother 70 2992–2999. 10.1093/jac/dkv220 26221017

[R17] Knight D. R. Hart J. Gottardo N. G. Eyre D. W. Crook D. W. Riley T. V.(2015b). Two cases of *Clostridium difficile* infection in unrelated oncology patients attributable to a single clone of *C. difficile* PCR ribotype 126. JMM Case Reports 2.

[R18] Knight D. R. Squire M. M. Riley T. V.(2015c). Nationwide surveillance study of *Clostridium difficile* in Australian Neonatal pigs shows high prevalence and heterogeneity of PCR ribotypes. Appl Environ Microbiol 81 119–123. 10.1128/AEM.03032-14 25326297 PMC4272713

[R19] Knight D. R. Riley T. V.(2016). *Clostridium difficile* clade 5 in Australia: antimicrobial susceptibility profiling of PCR ribotypes of human and animal origin. J Antimicrob Chemother 71 2213–2217. 10.1093/jac/dkw124 27130808

[R20] Lessa F. C. Mu Y. Bamberg W. M. Beldavs Z. G. Dumyati G. K. Dunn J. R. Farley M. M. Holzbauer S. M. Meek J. I.(2015). Burden of *Clostridium difficile* infection in the United States. N Engl J Med 372 825–834. 10.1056/NEJMoa1408913 25714160 PMC10966662

[R21] McDonald L. C. Coignard B. Dubberke E. Song X. Horan T. Kutty P. K. Ad Hoc *Clostridium**difficile* Surveillance Working Group(2007). Recommendations for surveillance of *Clostridium difficile*-associated disease. Infect Control Hosp Epidemiol 28 140–145. 10.1086/511798 17265394

[R22] Moono P. Putsathit P. Knight D. R. Squire M. M. Hampson D. J. Foster N. F. Riley T. V.(2016). Persistence of *Clostridium difficile* RT 237 infection in a Western Australian piggery. Anaerobe 37 62–66. 10.1016/j.anaerobe.2015.11.012 26679487

[R23] O'Connor J. R. Galang M. A. Sambol S. P. Hecht D. W. Vedantam G. Gerding D. N. Johnson S.(2008). Rifampin and rifaximin resistance in clinical isolates of *Clostridium difficile*. Antimicrob Agents Chemother 52 2813–2817. 10.1128/AAC.00342-08 18559647 PMC2493101

[R24] Rupnik M.(2007). Is *Clostridium difficile*-associated infection a potentially zoonotic and foodborne disease? Clin Microbiol Infect 13 457–459. 10.1111/j.1469-0691.2007.01687.x 17331126

[R25] Rupnik M. Janezic S.(2016). An update on *Clostridium difficile* toxinotyping. J Clin Microbiol 54 13–18. 10.1128/JCM.02083-15 26511734 PMC4702747

[R26] Slimings C. Armstrong P. Beckingham W. D. Bull A. L. Hall L. Kennedy K. J. Marquess J. McCann R. Menzies A.(2014). Increasing incidence of *Clostridium difficile* infection, Australia, 2011-2012. Med J Aust 200 272–276. 10.5694/mja13.11153 24641152

[R27] Squire M. M. Carter G. P. Mackin K. E. Chakravorty A. Noren T. Elliott B. Lyras D. Riley T. V(2013). Novel molecular type of *Clostridium difficile* in neonatal pigs, Western Australia. *Emerg**Infect Dis* 19 790–792. 10.3201/eid1905.121062 PMC3647499 23697508

[R28] Weese J. S.(2010). *Clostridium difficile* in food-innocent bystander or serious threat? Clin Microbiol Infect 16 3–10. 10.1111/j.1469-0691.2009.03108.x 20002685

